# Product policy - the main component of the marketing mix in the Romanian health services

**Published:** 2016

**Authors:** BI Coculescu, VL Purcarea, EC Coculescu

**Affiliations:** * “Titu Maiorescu” University, Faculty of Medicine; Centre of Preventive Medicine, Ministry of National Defense, Bucharest, Romania; ** “Carol Davila” University of Medicine and Pharmacy, Faculty of Medicine, Bucharest, Romania; *** “Carol Davila” University of Medicine and Pharmacy, Faculty of Dental Medicine, Bucharest, Romania

**Keywords:** product policy, marketing mix strategy, Romanian healthcare services

## Abstract

The objectives of the reforms in the EU healthcare systems are based on the implementation of the marketing concept in the health systems, which are, among other things:

• efficient management of the financial resources and control costs of the rendered health services;

• increased satisfaction of the clients of health care services;

• broad accessibility to health services;

• effective implementation of modern technologies;

• rational stimulation of medical services consumption;

• achievement of a fair and neutral competition between the public/ private providers and health insurance companies;

• introduction of performance criteria in order to increase the incomes of the medical staff and hierarchy in hospitals;

• implementation of modern management methods in health services management;

• decentralization of the public healthcare system.

Product policy in the medical system of healthcare - the most important component of the marketing mix - is the attitude that addresses a medical organization to the volume, structure, and diversity of services subject to their own activities in relation to the requirements of the services market and the competitive actions of other medical institutions.

## Introduction

The marketing concept can be expressed in terms of four general groups of instruments known as “the four Ps of marketing”: 1. Product, 2. Price, 3. Place (Distribution), and 4. Promotion (McCarthy). The 4 Ps represent the views of the seller on the marketing tools which are available, influence the buyer and can be translated into health services marketing by equivalence with the words: services, tariffs, access and retention. The Group of the four Ps of the seller corresponds to the 4 Cs of the client group (Lauterborn): 1. Consumer wants and needs, 2. Cost for the customer, 3. Convenience, and 4. Communication. The winners on the market will be those firms that can meet the customer needs in terms of economy and convenience for the customer as well as effective communication [**1**].

The important thing in distinguishing between the consumer goods marketing and services marketing is the increase of the marketing mix, the central element of the marketing strategy, the “4 Ps” to the “7 Ps”: Product, Price, Place, Promotion, Physical Evidence, Participants, and Process.

In order to strengthen its position in the healthcare system and cope with the difficulties caused by the competitive environment of other health institutions, state and private medical organizations require appropriate management policies [**2**].

The orientation towards one of the marketing policies with a major impact in the organizations providing healthcare services requires:

• a careful analysis of the needs and aspirations of customers; 

• the targeting of those patients whose needs can be achieved by the service organization through the existing resources at the respective health facility; 

• the finding of the most effective way of achieving benefits associated with reduced costs to maximize profits; 

• the placing of the offers required by the patients on the market for medical services; 

• as well as the prompt reaction and action to the changes of health services market which is constantly evolving through a flexible organization and functioning structure, connected to the financial needs of the patients [**2**,**3**,**4**,**5**]. 

## Discussion

In medical marketing, the product consists of what is sold (offered) to the healthcare consumer, namely patients, the expected behavior of health providers and benefits associated with this behavior. It appears as a global product consisting of a series of uniform items that can be grouped into: basic items expected or desired and improved - each giving the overall product specific characteristics [**2**]. 

**Fig. 1:**
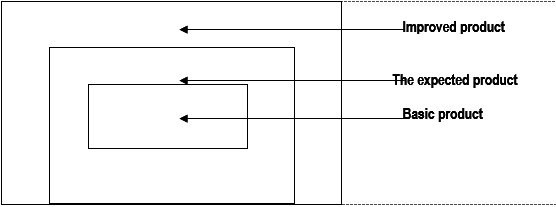
**Fig. 1**The three levels of global product in marketing healthcare services

In order to establish the marketing strategies it is necessary to identify the three levels of the overall health care product (**Fig. 1**).

The global health care product is prepared and implemented according to the needs and wishes of a selected group of patients, well defined and established by the audiences.

The basic product represents the main activity of the medical organizations, the reason of “being” of health care. It answers a series of questions:

▪ What does the product contain in order to be purchased by patients? 

▪ What are the benefits that patients receive? 

▪ What are the needs of the health care consumer the product meets? 

▪ Does the product solve the consumer’s problems? [**6**,**7**]. 

The expected or desired product, representing the next level of the global product also involves the patient’s participation by the minimum conditions that the latter expects from health providers. The expected product contains the conditions necessary for the patient to receive benefits identified in the core product, namely additional conduct (e.g. the hospitalized patient wants to have civilized hotel conditions, to be given medical services promptly, to be made aware of any investigations and treatments that he/ she is to receive, etc.).

An improved product includes both services promoted by marketers and tangible objects associated with the product expected to highlight the advantages that create a medical organization in order to increase the patient’s addressability and get the reputation of the institution.

Successful organizations do not respond only to the expected medical needs of the patients, they succeed in overcoming them through the granted facilities. Therefore, consumers of high quality health care services consider the global service a benefit package that will meet their needs not just a set of attributes. For example, there are leaflets and magazines that promote a healthy lifestyle in the waiting room in a medical office.

Currently, competition generally manifests itself in the improved product, successful organizations manage to provide advantages that not only satisfy, but also delight the audience. Since the advantages specific to the last level of the product structure tend to be benefits expected by patients, healthcare organizations must identify competitive advantages and new features to highlight their offer [**1**,**6**].

The main objectives of the health organizations aiming the product policy are the following:

• establishing a better position in the healthcare market; 

• presenting a better offer compared to the other organizations; 

• positioning compared to different types of patients; 

• increasing the number of patients assigned to the medical unit; 

• developing and implementing a strategy to balance the patient oriented actions and marketplace oriented ones. 

The strategic options regarding the product policy are based on three criteria: 

1.Quality

2. Productivity 

3. Degree and diversity [**6**,**7**]

Quality holds a dominant position compared to the other two criteria because in marketing, the services offered are regarded as “quality makes the difference”, therefore it is considered the key characteristic of health services.

The continuous growth of quality in the healthcare services is a strategic objective difficult to achieve, which means attracting and keeping both patients and healthcare professionals involved in the provision of healthcare services. This mission implies the fulfilling of the expectations of patients and the provision of basic services at a high level.

It is important that the provided medical service meets the needs of patients in an objective manner and even exceed these requirements, being at a higher level. Medical staff quality - accuracy, timeliness, professionalism, reputation medical organization (hospital, clinic, doctor, etc.) - and the degree of attachment of the patient are factors that influence the quality of service delivery, the optics patients.

In order to be in a competitive position in the competitive market of health services, health care organizations must offer a price as low as possible and at the same time seek to increase productivity. This objective, which is to remain in a favorable position on the market, can be achieved by adopting one of the following strategies: continuous training of medical personnel in the organization, improving and perfecting the healthcare services through the acquisition of technology and modern equipment, achieving the standard cost of medical services, increased addressability of patients by increasing the number of health care services provided, etc.

## Conclusions

Provision of medical care as a whole is the central point for any medical organization in implementing the marketing policy.

Therefore, we consider the assertion of marketing in healthcare [**5**,**6**,**7**,**8**] appropriate and necessary, implying the following:

• adopting of medical theory and practice, of important concepts in marketing; 

• developing new concepts and improving the existing ones by other notions arising from medical practice; 

• transposition into theory and continuous deepening of concepts arising from practice; 

• testing new methods of investigating the provision of healthcare services and behavior of patients; 

• designing reliable elements of forecasting health services market developments. 

For the reorganization of the health system, the desire to increase the efficiency in the provision of healthcare quality services and increasing presence of the private sector in healthcare are necessary organizational measures involving inter alia training of personnel to ensure the management of the existing resources in accordance with the requirements of economic efficiency and those imposed by the specific medical system. 
